# Supplementary material for: Changes in Package Sizes of Savoury Snacks through Exploration of Euromonitor and Industry Perspectives
Source: Int J Environ Res Public Health. 2022 Jul 30;19(15):9359. doi: 10.3390/ijerph19159359 (PMC9368387; doi:10.3390/ijerph19159359)
Supplement: Supplementary file 1 [file ijerph-19-09359-s001.zip › ijerph-1807192-supplementary.pdf]

Supplementary materials

Figure S1. Search tree for sales and package size statistics and qualitative data for savoury snacks in the Euromonitor database

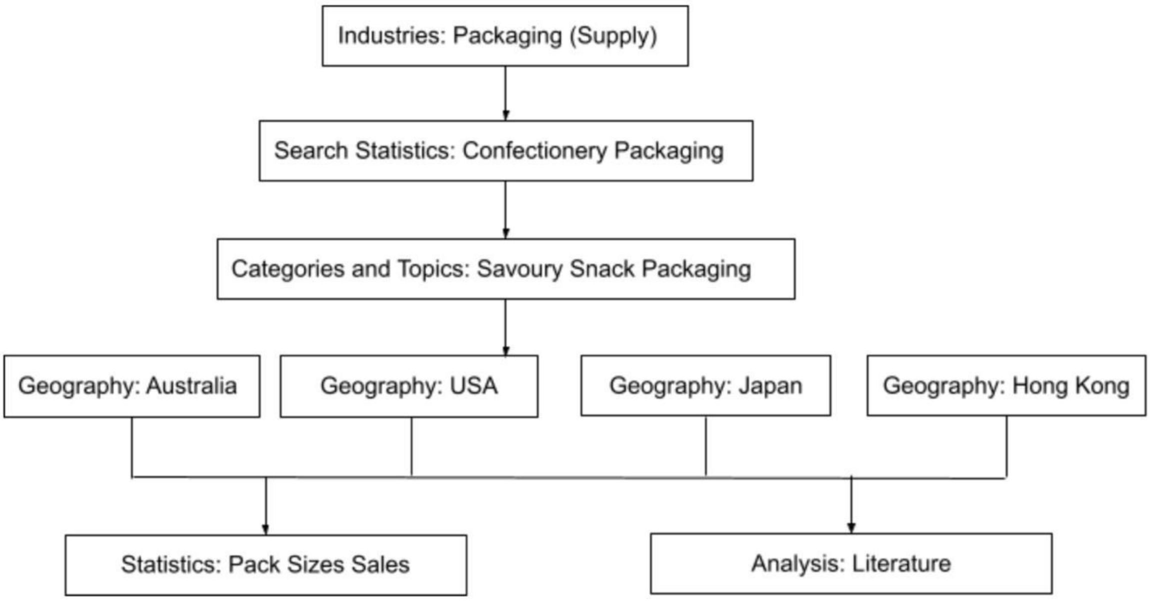

Figure S2. Percentage volume growth (CAGR) by snack category from 2016 - 2021 across Australia, the USA, Japan and Hong Kong

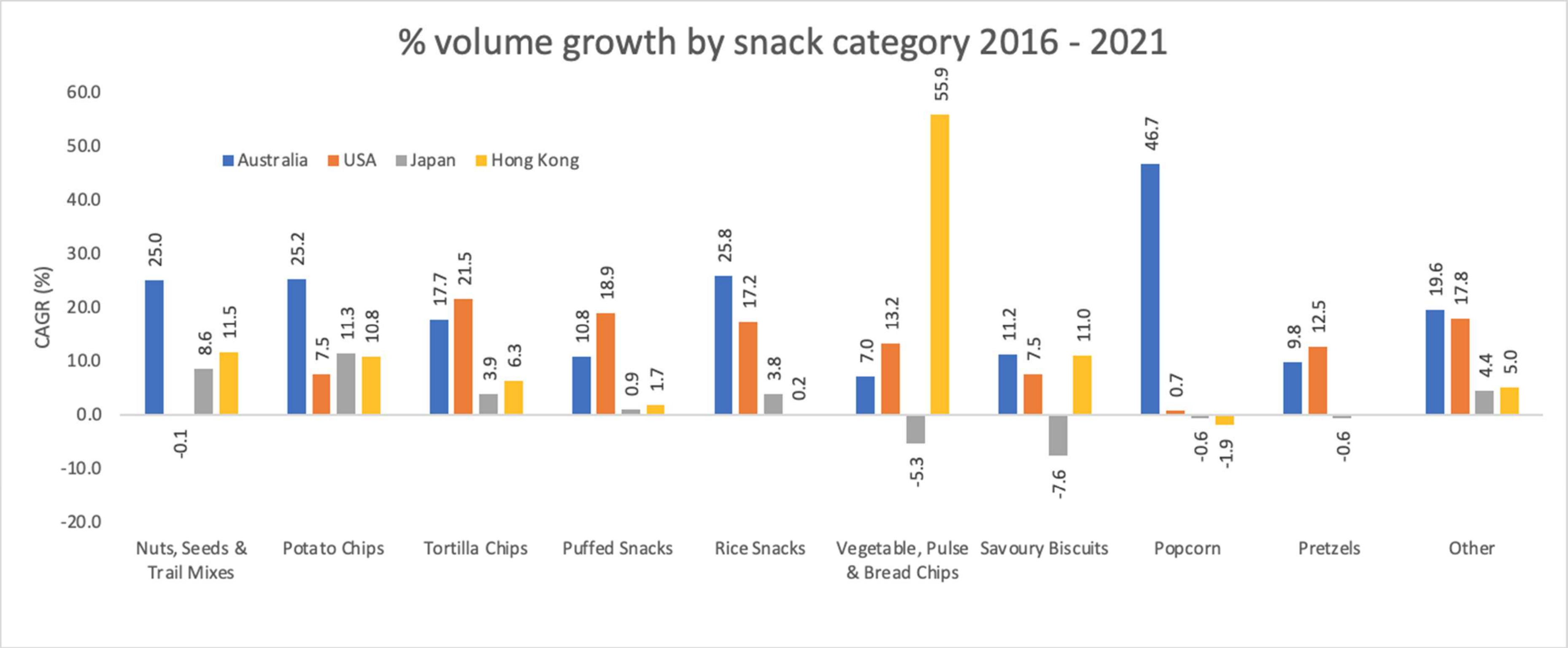

Table S1. Definitions and savoury snack subcategories on Euromonitor

Retail sales[1] definition	Sales through establishments primarily engaged in the sale of goods for home use and consumption.<br>Grocery retailers included grocery retailers, supermarkets, hypermarkets, convenience Stores, discounters, forecourt retailers, food/drink/tobacco specialists, independent small grocers and other grocery retailers. Other non-grocery specialists included health and beauty specialist retailers, and other foods non-grocery specialists.
Savoury Snack Categories	1. Nuts, Seeds and Trail Mixes<br>2. Salty Snacks<br>a. Potato Chips<br>b. Tortilla Chips<br>c. Puffed Snacks<br>d. Rice Snacks<br>e. Vegetable, Pulse and Bread Chips<br>3. Savoury Biscuits<br>4. Popcorn<br>5. Pretzels<br>6. Other Savoury Snacks[2]

[1]Only retail sales data was available for the savoury snacks category in Euromonitor.

[2]The category of "Other Savoury Snacks" differs across countries/regions.

- Australia: Meat jerky, seaweed snacks, noodle snacks;
- USA: Not specified;
- Japan: Dried fish and seafood snacks, seaweed snacks, meat snacks, pickled quail eggs snacks;
- Hong Kong: Seaweed snacks, seafood snacks, fish skin, jerky, scratchings, snack sausages, noodle snacks, snack egg

Table S2. Search terms for Medline database
Search topic: savoury snack package sizes

1	(savo?ry adj1 snack*).tw.
2	(salt* adj1 snack*).tw.
3	snack*.tw.
4	(junk adj1 food*).tw.
5	discretionar*.tw.
6	(discretionary adj1 food*).tw.
7	(packag* adj1 snack*).tw.
8	(packag* adj1 food*).tw.
9	chips.tw.
10	crisps.tw.
11	cracker*.tw.
12	biscuit*.tw.
13	popcorn*.tw.
14	pretzel*.tw.
15	nuts.tw. 6391
16	(trail adj1 mix).tw.
17	(tortilla adj1 chip*).tw.
18	(puff* adj1 snack*).tw.
19	(rice adj1 snack*).tw.
20	(veg* adj1 chip*).tw.
21	(pulse* adj1 snack*).tw.
22	(bread adj1 chip*).tw.
23	(meat* adj1 jerky).tw.
24	(roast* adj1 seaweed*).tw.
25	(noodle adj1 snack*).tw.
26	1 or 2 or 3 or 4 or 5 or 6 or 7 or 8 or 9 or 10 or 11 or 12 or 13 or 14 or 15 or 16 or 17 or 18 or 19 or 20 or 21 or 22 or 23 or 24 or 25
27	(pack* adj1 siz*).tw.
28	(container* adj1 siz*).tw.
29	(bag* adj1 siz*).tw.
30	(box* adj1 siz*).tw.
31	(carton* adj1 siz*).tw.
32	(multi adj1 pack*).tw.
33	(sale* adj2 trend*).tw.
34	(market adj2 snack*).tw.
35	(siz* adj2 increas*).tw.
36	(siz* adj2 decreas*).tw.
37	packag*.tw.
38	(packag* adj1 siz*).tw.
39	27 or 28 or 29 or 30 or 31 or 32 or 33 or 34 or 35 or 36 or 37 or 38
40	Australia.mp.
41	Japan.mp.
42	Hong Kong.mp.
43	USA.mp.
44	America.mp.
45	40 or 41 or 42 or 43 or 44
46	26 and 39 and 45

Table S3. Search terms for Proquest Central database
Search topic: savoury snack package sizes

(noft(("savo?ry NEAR/1 snack*") OR ("salt* NEAR/1 snack*") OR ("snack*") OR (junk NEAR/1 food*) OR (discretionar*) OR ("discretionary NEAR/1 food*") OR ("packag* NEAR/1 snack*") OR ("packag* NEAR/1 food") OR (chips) OR (crisps) OR (cracker*) OR (biscuit*) OR (popcorn*) OR (pretzel*) OR (nuts) OR ("trail NEAR/1 mix*") OR ("tortilla NEAR/1 chip*") OR ("puff* NEAR/1 snack*") OR ("rice NEAR/1 snack*") OR ("veg* NEAR/1 chip*") OR ("pulse* NEAR/1 snack*") OR ("bread NEAR/1 chip*") OR ("meat NEAR/1 jerky") OR (roast* NEAR/1 seaweed) OR ("noodle NEAR/1 snack*")) AND noft(("pack* NEAR/1 siz*") OR (container* NEAR/1 siz*) OR (bag* NEAR/1 siz*) OR (box* NEAR/1 siz*) OR (carton* NEAR/1 siz*) OR (multi NEAR/1 pack*) OR (sale* NEAR/2 trend*) OR (market NEAR/2 snack*) OR (pack* NEAR/1 siz* NEAR/2 increas*) OR (pack* NEAR/1 siz* NEAR/2 decreas*))) AND noft((Australia) OR (USA) OR (America) OR (Japan) OR (Hong Kong))

Table S4. Search words for grey literature (google search engine and company reports and websites)[3]

Search Topics	Search words
1. Savoury Snacks and Packaging Sizes	Savoury snacks package sizes OR salty snacks package sizes OR savoury snacks pack sizes OR salty snacks pack sizes OR PepsiCo OR Unisnack OR Smiths OR Frito-Lay OR Calbee OR Kraft OR sizes increase OR sizes decrease OR larger sizes OR smaller sizes
2.Savoury Snack Companies	Australia Companies:<br>1. Smiths Snackfoods Co Pty Ltd, The<br>2. Arnott's Biscuits Ltd<br>3. Coles Group Ltd<br>4. Woolworths Group Ltd<br>5. Snack Brands Australia<br>6. Menora Foods Pty Ltd<br>7. Aldi Stores Supermarkets Pty Ltd<br>8. Calbee Australia Pty Ltd<br>9. Stuart Alexander and Co Pty Ltd<br>10. Sakata Rice Snacks Australia Pty Ltd<br><br>USA Companies:<br>1. Frito-Lay Co.<br>2. Mondelez International Inc.<br>3. Campbell Soup Co<br>4. Keebler Foods Co.<br>5. Link Snacks Inc.<br>6. ConAgra Brands Inc<br>7. Kellogg Co<br>8. Kraft Heinz Co<br>9. Wonderful Co LLC, The.<br>10. Utz Quality Foods Inc.<br><br>Japan Companies:<br>1. Calbee Foods Co Ltd<br>2. Natori Co Ltd<br>3. Sanko Seika Co Ltd<br>4. Kameda Seika Co Ltd<br>5. Iwatsuka Confectionary Co Ltd<br>6. KOIKE-YA Inc<br>7. Kuriyama Beika Co Ltd<br>8. Yamazaki Biscuits Co Ltd<br>9. Denroku Co Ltd<br>10. Kellog (Japan) GK<br><br>Hong Kong Companies:<br>1. Kraft Foods Hong Kong Ltd<br>2. Calbee Four Seas (HK) Co Ltd<br>3. PepsiCo Inc<br>4. Four Seas Mercantile Holding Ltd<br>5. Garden Co Ltd, The

	6. URC HK Co Ltd<br>7. Kellogg Co<br>8. Haitai Confectionery Co Ltd PT<br>9. Wah Yuen Food Co Ltd<br>10. TaoKaeNoi Food & Marketing Co Ltd

[3]The two search topics were investigated in two separate searches. The first search topic of interest (Savoury Snacks and Packaging Sizes) was limited to the first 100 search results for screening. The second search topic of interest (Savoury Snack Companies) was done by searching for websites and official reports for each of the top 10 manufacturing companies of each country/region.

Table S5.1. Database search results for industry reasons for change in package sizing of savoury snacks (Medline and Proquest Central)

Country/ Region	Year	Article Type	Title	Direction of Change	Results	Reasons for change
USA	2009	Journal Article	Variety Packs [56]	Decrease	To respond to changing environments and consumer demands for portion control and convenience, snack producers are rolling out their own baked, all-natural and grab-and-go snacks.<br>Consumers are demanding more 100-calorie packs for portion control.	Portion control<br><br>Convenience<br><br>Increasing profit and product line expansion
USA	2019	Journal Article	Sealing The Deal [24]	Decrease	Millennials have demonstrated a preference for single-portion snack foods over traditional sit- down meals, and the number of one-person households is rising. As a result, bag sizes have gradually decreased over the years. "In fact, 100-calorie, bite-size snack bags are now ubiquitous in grocery aisles. For many manufacturers, reducing the size of their bags is the best way to offer consumers convenience and calorie control without jeopardizing brand loyalty".	Snacking as meal replacement<br><br>Portion control<br><br>Convenience

Table S5.2. Grey literature search results (including Google search results, Euromonitor reports and company reports and websites) for industry reasons behind changes in package sizing of savoury snacks

Country/ Region	Year	Article Type	Title	Direction of Change	Results	Reasons for change
**Google Search Results**						
Global	2020	Webpage	Snack Industry Overview [57]	Increase	Producers are adapting to evolving environments, especially seen in the COVID-19 pandemic. Larger package sizes and multi-packs have seen an increase in sales due to COVID-19 snacking behaviours.	COVID-19 snacking behaviour
Global	2015	News Article	Global savory snacks to hit $166.6bn by 2020 [9]	Decrease	The global savoury snack market is expected to grow by 7.1% each year and there has been an increasing demand for healthier snack options. Snack manufacturers have been responding to these demands for portion control by producing smaller pack sizes and this trend has been growing across the global snack market. Furthermore, lower-income groups who cannot afford larger packs also drive sales and production of smaller packs.	Health consciousness trends<br>Portion control<br>Affordability issues
Global	2016	Report	Smaller is Better as Global Packaging Growth is Shaped by Variation in Pack Sizes [58]	Decrease	A shift to smaller pack sizes is the trend seen in packaging volume growth and is known as “rightsizing”. Snacking, portion control and consumer mindfulness are the main driving factors behind the movement to buy in smaller quantities. These trends are also supported by a number of government initiatives that address national health concerns. Affordability is another factor that strengthens the success of the ongoing smaller pack size trend.	Health consciousness trends<br>Portion control<br>Affordability issues<br>International recommendations
Global	2020	News Article	Produce-based	Increase	IRI reports 92% of millennials and Gen Z members replace at	Snacking as meal

			snack foods on the rise [59]		least one meal per week with snacks, while 7% do not eat traditional meals anymore. This trend has resulted in a want for larger package sizes, as well as multipacks and more variety packs.<br>While 4.5- to 8-ounce package sizes is still the largest seller as it was pre-COVID, the 10- to 16-ounce category has seen the largest increase.	replacements
USA	2019	Newsletter	Doritos unveils new variant and larger 230g bags [60]	Increase	Doritos is expanding its range of tortilla chips with the addition of bigger pack sizes.<br>“We have had excellent results from our consumer testing, with 86% of consumers saying that they would buy the product”.	Increasing profit and product line expansion
USA	2017	Webpage	Downsizing to compete with snacks [61]	Decrease	Changes in lifestyle have led to an increased demand for portability and consuming snacks instead of 3 meals a day. Smaller pack sizes for portion control continue to be increasing in popularity in the baking and snacking industries not only due to its perceived healthy attributes but also for its convenient and portable characteristics.	Health consciousness trends<br><br>Portion control<br><br>Snacking as meal replacement<br><br>Convenience
USA	2016	Webpage	Hummus Plus Chicken Adds Smaller Snacking Sizes [62]	Decrease	Snacking continues to be a megatrend in the U.S., with consumers looking for smaller portions in on-the-go packaging. The new Hummus Plus snack-size package delivers on many fronts, with portability, nutrition and convenience	Convenience<br><br>Portion control

USA	2018	Journal	PepsiCo cuts sodium content in Lay’s, shrinks pack sizes of 2 salty snacks [63]	Decrease	PepsiCo has resized the packaging for Lay’s and Kurkure snacks and reduced the consumption of paper used in cartons, which will lower fuel consumption and shrink its carbon footprint.	Corporate responsibility
USA	2021	Webpage	Packaging Trends 2021 [46]	Increase	Consumers shift from single-serve formats to multipacks and larger pack sizes for better value for money. There are also new snacking occasions made as consumers spend more time at home and work from home due to COVID-19. The social times within the household including snack-sharing behaviour are valued.<br>“During stockpiling in early 2020 alone, the 10 to 16-oz snacks pack size bracket in the US (283g-453g) grew the most with an 87% increase in US$.”	Value for money<br><br>COVID-19 snacking behaviour<br><br>Social sharing behaviour
USA	2017	Webpage	Demand for unique snacks breeds creativity in a single-serve packaging [64]	Decrease	Consumers are appealed to individually or small packaged goods for convenience and portability, as seen with baked goods and bars. There is an increasing demand for savoury snacks and crackers to also be produced in smaller single-serve packaging that can be taken on the go and eaten entirely before throwing the package away.	Convenience<br><br>Health consciousness trends
USA	2021	Webpage	Experts predict post-pandemic flavor trends [65]	Decrease	Conagra Brands Inc (Chicago) and other savoury snack companies are offering more varied pack sizes for many meat snacks. Larger formats are for consumers following a low-carb lifestyle, while smaller sizes satisfy savory cravings during the day.	Health consciousness trends

USA	2020	News Article	The state of the snack industry in 2020 [47]	Increase	COVID-19 has greatly changed snacking behaviour amongst consumers. There has been a general trend toward more indulgence, including potato chips, tortilla chips and cheese snacks and the purchase of larger sizes and/or multi-packs for convenience and value for money as families are making less trips to the grocery store.<br><br>"...sales spiked for all pack sizes, but particularly for larger package sizes...the 10 to 16-oz pack sizes grew the most with an 87% increase in dollar sales … and larger than 16-oz increasing sales by double digits."	COVID-19 snacking behaviour<br><br>Social sharing behaviour<br><br>Convenience<br><br>Value for money
USA	2020	Webpage	How the latest in food processing and packaging technology can help sustain consumer appetite for savory snacks [66]	Decrease	The World Health Organization (WHO) has recommended that governments regulate food advertising and marketing to change perceptions of 'normal' portion size and healthy food options. Smaller packaging sizes and single-serve portions are therefore on the rise, appealing to those looking to limit calorie intake and restrict portion sizes.<br><br>"As a result of the crackdown on portion sizes, the way snacks are packaged is changing – with a shift from larger share-size bags to single portion packets."	International recommendations<br><br>Health consciousness trends<br><br>Portion control

USA	2020	Webpage	Snack food industry trends: Small is the new big in snack packaging [67]	Decrease	COVID-19 is a health crisis that presents an additional mental strain on consumers who need extra assurance that their favorite snacks are safe to eat. This has led to an increase in single-serve packaged products as it was seen as more sanitary because of limited access to package contents. Previously, bulk snack food packaging was the standard. Consumers expected to portion out products on their own and felt buying in bulk was the frugal and responsible choice, especially for families. However, today's emerging generations desire convenience and portability above all, and this is especially evident when exploring current snack food industry trends.	Health consciousness trends<br><br>COVID-19 snacking behaviour<br><br>Convenience
USA	2020	Webpage	WATCH: Consumer behavior post the coronavirus ‘surge’... What to expect over the next three to four weeks, IRI, BCG [68]	Increase	There is an expectation for continued shifts to larger pack sizes across product categories as people replace on-premise and away-from-home consumption with at-home consumption during COVID-19 pandemic	COVID-19 snacking behaviour

USA	2021	Webinar	How America Eats: The Snacking Frenzy in 2021. [45]	Increase	Due to COVID 19, there has been less "on-the-go" movement which has seen a shift to larger pack sizes of snack and a decline in smaller pack sizes in units and volume of sale. Due to pandemic restrictions, 26% of individuals are consuming more indulgent snacks more often as comfort food. However, it is forecasted that as consumers become mobile again post-pandemic, smaller pack sizes will rebound as well.	COVID 19 snacking behaviour<br><br>Social sharing behaviour
USA	2021	Webpage - blog post	How the Pandemic Has Forced the Snack Industry to Evolve [44]	Increase	The COVID-19 pandemic has prompted consumers to eat more snacks, more often. Mondelez's annual State of Snacking study reported 52% of adults around the world agreed that snacking has been a "lifeline" throughout the pandemic, and that 88% of adults are snacking more or the same during the pandemic as previously.<br>There has been a complete reversal of the prevailing trend before March 2020 where smaller snack pack ("on-the-go") portion sizes had been growing in popularity for a number of years to cater to consumers' increasingly active lifestyles. With COVID-19 reducing activity levels, larger package sizes and multipacks have seen a resurgence.<br>To appeal to consumers, manufacturers have focused on:<br>- Producing healthier snacks with more nutritional value<br>- Ensuring more of their products are available through ecommerce<br>- Selling products in larger, better value pack sizes	COVID-19 snacking behaviour<br><br>Health consciousness trends<br><br>Value for money

USA	2019	Webpage	Smaller Pack Sizes Drive Sales for PepsiCo [69]	Decrease	"Convenience and snacking trends are driving consumer demand for snacks in smaller packages" PepsiCo CEO Ramon Laguarta<br><br>There has been a trend towards smaller packs due to smaller households and more occasion-based purchases.	Convenience
USA & UK	2015	Journal Article	Portion Size: What we know and what we need to know [48]	Increase and decrease	One response to the widespread concern about large portion sizes, and their role in obesity, has been for industry to offer products in a variety of different sized packages. This approach assumes that the offering of food items in smaller quantities allows the consumer to exercise better self-restraint.<br><br>However, a major disincentive to buying smaller packages is that better value is perceived when buying in bulk.	Health consciousness trends<br><br>Value for money
Australia & NZ	2015	Government background paper	Food and Nutrition Guidelines for Healthy Children and Young People (Aged 2 –18 years) A background paper [70]	Increase	Larger portion sizes often have a lower price per unit, so appear to be better 'value for money'.	Value for money

Euromonitor Reports						
USA	2021	Report	Savoury Snacks in the US [71]	Decrease	With the increasing health consciousness trend, consumers are attracted to products that are baked rather than fried or popped. This has seen an increase in the rice snacks and popcorn categories in particular, being lighter in weight than other snacks, fostering the perception amongst consumers that they are eating less.	Health consciousness trends
Australia	2021	Report	Savoury Snacks in Australia [32]	Decrease	Health and wellness trends continue to drive new product development in savoury snacks as consumers opt for more natural and lower fat products in 2021 (eg. popcorn) Smaller pack sizes have seen the greatest increase in volume production and sales.	Health consciousness trends
Japan	2021	Report	Savoury Snacks in Japan [72]	Decrease	COVID-19 continues to have a positive impact on sales of some savoury snacks such as potato chips in 2021. Japan has seen a large growth in potato chips and nuts, seeds and trail mixes with the latter likely due to increased health consciousness trends.	Health consciousness trends
Hong Kong	2021	Report	Savoury Snacks in Hong Kong [33]	Decrease	Nuts, seeds and trail mixes offer consumers a natural source of protein in smaller packaging, in line with the on-the-go consumption trend. Leading savoury snacks brand Calbee will continue to target health-conscious consumers with baked chips positioned as healthy, vegan and gluten-free options that are also GMO-free and low in sugar and salt.	Health consciousness trends<br><br>Convenience
**Company Reports and Relevant Websites**						

Country/ Region	Year	Article Type	Company Name	Direction of Change	Results	Reasons for change
USA	2021	Webpage	Frito-Lay Co. [31]	Decrease	The most important thing to consumers is convenience (which can come from smaller pack sizes) or having tried-and-true favourites.	Convenience
USA	2021	Company report	Mondelez International Inc. [29]	Decrease	It is expected that 20% of global snacks net revenue will be from portion control products by 2025. This is due to increased health consciousness and consumers leading busier lifestyles. Consumers increasingly want to feel good about their snacks and know that they are not harming the planet. Due to this, Mondelez has continued to renovate and innovate their portfolio to satisfy consumers' current and changing well-being needs and wants, with offers such as Better for You, Authentic Natural and Simple, Functional Nutrition and Permissible Indulgence. Mondelez also aims to provide less than 200 kcals per portion, smaller packages	Increasing profit and product line expansion<br><br>Portion control<br><br>Health consciousness trends<br><br>Corporate responsibility
USA	2021	Webpage	Link Snacks Inc. [74]	Decrease	Producing more meat snacks that are low in fat with only 80 calories and 180 mg sodium per 1oz package in alignment with increasing health consciousness trends.	Health consciousness trends
Japan	2018	Company Report	Calbee Inc. [41]	Decrease	Calbee is looking to expand the range of package sizes to entice customers wanting to try products. In addition to the regular large-size packages, Calbee produced smaller-size packages and single-servings (50g) to promote purchase by new customers	Increasing profit and product line expansion
Japan	2019	Company Report	Calbee Inc. [42]	Decrease	Calbee saw sales of domestic snack foods increased by 2.8%. The introduction of small size and individual portion size bags was used as a means to generate new domestic customers.	Increasing profit and product line expansion

Australia	2017	News Article	Arnotts Biscuits Ltd [28]	Decrease	Arnott's has shrunk the size of its popular products while keeping the prices the same. “As with all businesses, from time to time it is necessary for us to review the prices of our products. Recent increases in energy costs mean costs across our Australian bakeries are higher.”	Standardizing production & manufacturing costs
Australia	2015	Webpage	Smiths Snackfoods Co Pty Ltd, The [39]	Decrease	Smiths, owned by American food and beverage mega-giant PepsiCo, has reportedly said it needed to shrink the pack size to make up for higher manufacturing costs. PepsiCo’s Red Rock Deli potato chips fell from 185g to 165g per pack last year without a corresponding fall in price. Smith’s Potato Crisps and Doritos Corn Chips pack sizes went from 200g to 175g in 2010.	Standardizing production & manufacturing costs
Australia	2016	News Article	Stuart Alexander & Co Pty Ltd [40]	Decrease	The Daily Mail Australia reports that Pringles chips, sold in cardboard cylinders, have shrunk in size in Australia and are being sold in a deceptively similar package of the same height but with a smaller diameter. The shrinkage means that Pringles are now more expensive by stealth, from $2.70 per 100g to $2.98.	Standardizing production & manufacturing costs
